# Association between Severity of MERS-CoV Infection and Incubation Period

**DOI:** 10.3201/eid2203.151437

**Published:** 2016-03

**Authors:** Victor Virlogeux, Minah Park, Joseph T. Wu, Benjamin J. Cowling

**Affiliations:** Ecole Normale Supérieure de Lyon, Lyon, France (V. Virlogeux);; The University of Hong Kong, Hong Kong, China (V. Virlogeux, M. Park, J.T. Wu, B.J. Cowling)

**Keywords:** Middle East respiratory syndrome, Middle East respiratory syndrome coronavirus, MERS-CoV, coronavirus, viruses, incubation period, infection, illness, severity, South Korea

## Abstract

We analyzed data for 170 patients in South Korea who had laboratory-confirmed infection with Middle East respiratory syndrome coronavirus. A longer incubation period was associated with a reduction in the risk for death (adjusted odds ratio/1-day increase in incubation period 0.83, 95% credibility interval 0.68–1.03).

The incubation period of an infectious disease is the time from the moment of exposure to an infectious agent until signs and symptoms of the disease appear (*1*). This major biological parameter is part of the case definition and is used to determine duration of quarantine and inform policy decisions when mathematical modeling is used (*2*). Incubation periods vary from person to person, and their distribution tends to be right-skewed and unimodal (*3*). Variability in incubation periods for infection with Middle East respiratory syndrome coronavirus (MERS-CoV) has been described (*4*–*8*). Previous studies have not examined whether the length of the incubation period in a person has any correlation with subsequent clinical outcomes.

In 2015, South Korea had the largest outbreak of MERS-CoV infections outside the Arabian Peninsula (*6*). In a previous study, we reported that patients who died of severe acute respiratory syndrome (SARS) coronavirus infection had a shorter incubation period compared with infected patients who survived (*9*). The objective of this study was to examine the association between severity of MERS-CoV illness and length of incubation period.

## The Study

We retrieved publicly available data from the Korea Center for Disease Control and Prevention, the Korean Ministry of Health and Welfare, the World Health Organization, and local news reports in South Korea to compile a list of all confirmed cases that had been reported by July 26, 2015 (*6*). Exposure data were available for 109 (64%) of 170 patients. For most cases, information on exposure was recorded as intervals >2 days during which infection was believed to have occurred, rather than exact dates of presumed infection. For the subset of patients without available exposure data, we assumed that their incubation time was 0–21 days because 21 days was the longest incubation period reported (*9**,**10*). Data for patients is provided in Technical Appendix 1.

To estimate incubation period distribution, we fitted a gamma distribution that enabled interval censoring (*6*) by using Markov Chain Monte Carlo methods in a Bayesian framework (Technical Appendix 2) (*9*). In this analysis and analyses described below, we specified flat priors for each parameter and drew 10,000 samples from the posterior distributions after a burn-in of 5,000 iterations.

To evaluate potential factors, such as age and sex, that could be associated with length of incubation period, we fitted a multiple linear regression model to the data with the log incubation period as response variable and age and sex as explanatory variables. To determine the association between incubation period and severity of disease, we first estimated the difference in mean incubation period between patients who died and those who survived. However, this analysis could not account for potential confounders. Therefore, we specified a multivariable logistic regression model in which death was the binary response variable and predictors included age, sex, and the incubation time for each patient (*9*). We performed this analysis by using an exact likelihood approach and incubation times resampled from the 10,000 posterior samples in each iteration (Technical Appendix 2). All analyses were conducted by using R version 3.0.2 (R Foundation for Statistical Computing, Vienna, Austria). Raw data and R syntax enabling reproduction of results are available from the Dryad Digital Repository (http://dx.doi.org/10.5061/dryad.v3456).

Of 170 patients in this study, 36 (21%) died. Mean patient age was 54.6 years, and 98 (58%) were male. Patients who died were significantly older than patients who survived (68.9 years vs. 50.8 years; p<0.001). No differences regarding age, sex, and case-fatality risk were observed between patients with or without recorded exposure data. We estimated a mean incubation period of MERS-CoV in all 170 patients of 6.9 days (95% credibility interval [CrI] 6.3–7.5 days) by using a gamma distribution. Age and sex had no associations with incubation period.

The mean incubation period was 6.4 days (95% CrI 5.2–7.9 days) for 36 patients who died compared with 7.1 days (95% CrI 6.3–7.8 days) for 134 patients who survived (Figure). The difference in means was 0.62 days (95% CrI −0.99 to 2.04 days). In the multivariable logistic regression model, we found that a longer incubation period was associated with a marginally reduced risk for death (odds ratio 0.83/1-day increase in incubation period, 95% CrI 0.68–1.03/day) after adjustment for age and sex (Technical Appendix 2 Table 2).

**Figure:**
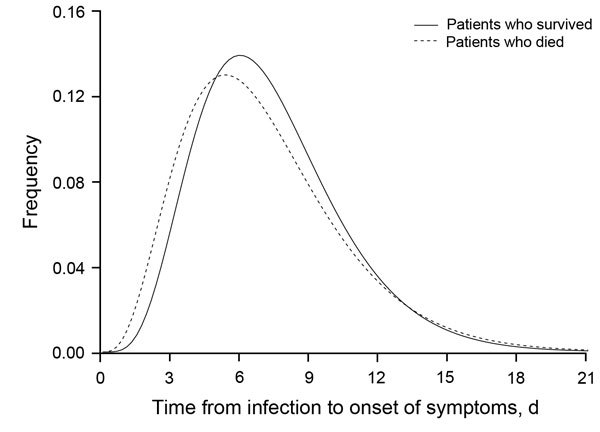
Parametric estimates of incubation period distribution for patients who died of infection with Middle East respiratory syndrome coronavirus (dashed line) and patients who survived infection (solid line), South Korea, 2015.

To examine sensitivity of our results, we also fitted the logistic regression models by using 3 categories for the incubation period. We observed similar results and a reduced risk for death associated with longer incubation periods (Technical Appendix 2 Table 2). Results were also consistent in the subset of 109 patients with recorded exposure intervals (Technical Appendix 2 Table 2).

## Conclusions

We estimated the incubation period of MERS-CoV cases during the recent MERS outbreak in South Korea and found that patients who died had a shorter incubation period than patients who survived. In a previous study, we found that the length of incubation period in patients infected with SARS coronavirus was also correlated with severity of the disease, with a shorter incubation period for patients who died (*9*). The pathogenesis of MERS-CoV and SARS coronavirus infection is similar (*11*), with a rapid progression to respiratory failure and intubation occurring ≈1 week after onset of symptoms and up to 5 days earlier in MERS patients than in SARS patients (*4*,*12*). Moreover, high rates of hemoptysis were observed in patients infected with MERS-CoV, which suggests severe lung injury (*4*).

MERS-CoV also has higher replication rates and shows broader cell tropism in the lower human respiratory tract than severe acute respiratory syndrome coronavirus (*13*). These results suggest that a shorter incubation period could be related to a higher initial infective dose and consequently to faster or greater pathogen replication. This finding could lead to a more severe disease induced by more aggressive and damaging inflammatory responses (*14*). Closer monitoring of patients who have a shorter incubation period could be considered during such outbreaks.

Another potential explanation for our findings is that patients with longer incubation periods were identified and infection confirmed more quickly. This improvement in time to identification and admission to a hospital led to improved prognosis. Although longer incubation periods were correlated with shorter delays from onset to laboratory confirmation, we did not find evidence of a strong mediating effect of delay from onset to laboratory confirmation on the risk for death. However, with the small sample size, there was limited statistical power to detect a small-to-moderate effect.

Our study had some limitations. Our estimates of the incubation period were based on self-reported exposure data, which could be affected by recall bias. Moreover, 61 patients (36%) included in our main analysis had missing exposure data, and inclusion in a Bayesian framework with a wide interval of 0–21 days was necessary. Both of these limitations could have reduced the statistical power of our study to identify an association. Finally, we did not have information on underlying medical conditions or the geographic location of cases, or the treatments that were given to cases, and these variables could have been associated with clinical outcomes.

In conclusion, we found an association between shorter incubation periods among patients with MERS-CoV infection and a higher risk of death subsequently, similar to the association previously reported for severe acute respiratory syndrome coronavirus (*9*). This association might occur because the duration of the incubation period is an early reflection of disease pathogenesis.

**Technical Appendix 1.** Data for patients infected with Middle East respiratory syndrome coronavirus, South Korea, 2015.

**Technical Appendix 2.** Additional details of statistical methods for analysis of patients infected with Middle East respiratory syndrome coronavirus, South Korea, 2015.
