# Supplementary material for: Association between Severity of MERS-CoV Infection and Incubation Period
Source: Emerg Infect Dis. 2016 Mar;22(3):526–8. doi: 10.3201/eid2203.151437 (PMC4766874; doi:10.3201/eid2203.151437)
Supplement: Supplementary file 2 — Technical Appendix 2. Additional details of statistical methods for analysis of patients infected with Middle East respiratory syndrome coronavirus, South Korea, 2015. [file 15-1437-Techapp-s2.pdf]

Article DOI: http://dx.doi.org/10.3201/eid2203.151437

Association between Severity of MERS-CoV Infection and Incubation Period

Technical Appendix 2

Additional Details of Statistical Methods

The incubation period of an infectious disease is the time from the moment of exposure to an infectious agent until signs and symptoms of the disease appear. If infection occurred at time X_i for the patient *i*, and symptom onset occurred at time Z_i, the incubation period is defined as $T_i = Z_i – X_i$. However, estimation of the incubation period is often complicated because infection events cannot be directly observed. If patient *i* reported that infection most likely occurred in a period of exposure between times L_i and U_i, where $L_i \le X_i \le U_i$, the incubation time therefore is bounded by the interval $(Z – U_i, Z – L_i)$. These data are a special type of survival data, and a convenient approach would be to reverse the time axis setting Z as the origin and X as the outcome time. Reversing the time axis is valid only when the density function for infection is uniform in chronologic time. This condition should be reasonable in the setting of Middle East respiratory syndrome coronavirus, with each exposure interval being relatively short.

To evaluate the incubation period distribution, we compared the goodness of fit of different parametric models (gamma, lognormal, Weibull, and exponential distributions) usually used to describe the incubation period distribution of infectious diseases. We found that the gamma distribution had the best Bayesian Index Criterion value. We assumed consequently a gamma distribution with parameters (k, θ) and probability density function

$$f(t_i) = \frac{{t_i}^{k-1} e^{-\frac{t_i}{\theta}}}{\Gamma(k)\theta^k}$$

We assumed that the incubation period distribution had different parameters among the nonfatal cases and the fatal cases, and we consequently estimated 2 different parameters (k, θ) of the gamma distribution using Markov Chain Monte Carlo (MCMC) methods. We compared the mean incubation period between these 2 groups by using the 10,000 posterior samples of each

couple of parameters (k, θ). We then considered 2 approaches to estimate the association between the risk for death (outcome) and the incubation period (explanatory variable).

Multiple Linear Regression

We evaluated the potential association between the length of the incubation period and the age and sex of patients in both subgroups (fatal and nonfatal cases) by using a multiple linear regression approach within a Bayesian framework, and we did not find a significant association (Table 3).

Approach 1: Exact Likelihood Approach

Let *f* and *F* be the pdf and cdf of the incubation period, assumed to be gamma distributed with parameters *k* and θ and stratified by clinical outcome (fatal and nonfatal cases). Let *P* be the probability of death, which we assume to be dependent on age (*g*), sex (*s*) and incubation period (*x*) as in logistic regression: (1)

$$P(g,s,x) = \frac{1}{1+\exp[-(\beta_0+\beta_1 g+\beta_2 s+\beta_3 x)]}$$

Depending if the case *i* had an exact exposure date (A_1) or an interval of exposure (A_2), we defined the probability of death q_i. If case *i* is in A_1, then the probability of death is simply $q_i = P(g_i, s_i, x_i)$. If case *i* is in A_2, then the probability of death is

$$q_i = \int_{x_i^L}^{x_i^U} P(g_i,s_i,x)\,\frac{f(x|k_i,\theta_i)}{F\left(x_i^U\middle|k_i,\theta_i\right)-F\left(x_i^L\middle|k_i,\theta_i\right)}\,dx$$

where $[x_i^L, x_i^U]$ is the range of incubation period for case *i* and where (k_i, θ_i) is the couple of parameters of the gamma distribution depending if the case *i* belongs to the fatal or nonfatal cases group.

We estimated $\theta = (\beta_0,\beta_1,\beta_2,\beta_3,k_{fc},\theta_{fc},k_{nfc},\theta_{nfc})$ simultaneously using MCMC and the following likelihood:

$$L(\theta) = \prod_{i\in A_1} f(x_i|k_i,\theta_i)\prod_{i\in A_2}\left[F\left(x_i^U\middle|k_i,\theta_i\right)-F\left(x_i^L\middle|k_i,\theta_i\right)\right]\prod_i {q_i}^{d_i}(1-q_i)^{1-d_i} \quad (2)$$

where d_i = 1 if case i died from the disease and 0 otherwise; (k_{fc}, θ_{fc}) and (k_{nfc}, θ_{nfc}) are the 2 parameters of the gamma distribution for the fatal and the nonfatal cases, respectively.

Approach 2: Resampling Approach

We also defined a logistic regression model by using incubation times resampled from the 10,000 posterior samples. This approach enabled us to simulate the distribution with imputed values for individual incubation periods, which was particularly useful for an analysis in which we stratified incubation periods into tertiles. In general, the likelihood based approach might be preferred to this simulation approach, and we presented the simulation approach results as sensitivity analyses.

In this approach, the probability of death was similarly defined as in equation (1) and for each patient with interval-censored exposure data, we estimated 10,000 posterior samples for the incubation time by using MCMC, and we used the same likelihood as defined in equation (2), but where $q_i = P(g_i, s_i, x_i)$ for all cases, using the resampled incubation time for patients with interval-censored data.

Bayesian Framework

We used a Bayesian framework to estimate the different parameters of the logistic regression. In this framework, if θ represents a vector of parameters and y the data, and Bayes theorem gives us the following relationship:

$$p(\theta|y) = \frac{p(y|\theta)p(\theta)}{p(y)}$$

where $p(\theta)$ is the prior probability of the parameters θ , $p(y|\theta)$ is the likelihood function and $p(\theta|y)$ is the posterior probability of θ given the data y. The MCMC process was initiated by giving random values to the parameters θ and by choosing noninformative prior (flat prior) for θ. A Metropolis Hastings algorithm was used to update the parameter values in each iteration. In each iteration, all the k parameters are randomly generated using the normal distribution with the mean θ_k^{j-1} (previous value of the kth parameter) and standard error σ_k, $N(\theta_k^{j-1}, \sigma_k)$ for each parameter. The updated likelihood is compared with the previous one using the following accept-reject method:

$$q = \frac{p(y|\theta^j)p(\theta^j)}{p(y|\theta^{j-1})p(\theta^{j-1})}$$

If $q \geq 1$, the proposed new values of parameters θ^j are accepted. If $q < 1$, then θ^j values are accepted with probability q.

A burn-in period with 5,000 iterations was used to reduce the bias of the choice of the initial parameter values and to generate values only in the stationary distribution. The above algorithm was repeated 10,000 times after the burn-in period, with an acceptance rate included in [0.45, 0.55] for each parameter (adjusting on σ_k).

Technical Appendix Table 1. Characteristics of patients with cases of infection with MERS-CoV, South Korea*

Patient characteristics	Fatal cases	Nonfatal cases	Overall	p value
All patients				
Sample size, no. (%)	36 (21)	134 (79)	170 (100)	–
Mean ± SD age, y	68.9 ± 10.0	50.8 ± 15.4	54.6 ± 16.2	<0.001
Male sex, no. (%)	24 (67)	74 (55)	98 (58)	0.297
Mean incubation period, d (95% CrI)	6.4 (5.2–7.9)	7.1 (6.3–7.8)	6.9 (6.3–7.5)	–
Patients with recorded exposure intervals				
Sample size, no. (%)	26 (24)	83 (76)	109 (100)	–
Mean ± age, y	68.6 ± 10.0	50.4 ± 14.6	54.8 ± 15.7	<0.001
Male sex, no. (%)	18 (69)	47 (57)	65 (60)	0.361
Mean incubation period, d (95% CrI)	6.4 (5.2–8.0)	7.1 (6.4–7.8)	6.9 (6.3–7.5)	–

*MERS-CoV, Middle East respiratory syndrome coronavirus; –, not applicable; CrI, credibility interval.

Technical Appendix Table 2. Factors associated with risk for death from infection with MERS-CoV, South Korea*

	Risk for death,† OR (95% CI)	
Factors	All patients, n = 170	Patients with recorded exposure intervals, n = 109
Approach 1: continuous incubation period using exact likelihood		
Incubation period (continuous)	0.83 (0.68–1.03)	0.91 (0.75–1.10)
Age, y	1.11 (1.07–1.16)	1.13 (1.09–1.19)
Sex, M vs. F	2.24 (0.89–6.00)	3.15 (0.98–10.10)
Approach 2: continuous incubation period using resampling method		
Incubation period‡ (continuous)	0.81 (0.66–0.98)	0.91 (0.73–1.12)
Age, y	1.11 (1.08–1.16)	1.15 (1.09–1.23)
Sex, M vs. F	1.89 (0.73–5.32)	3.56 (1.02–13.86)
Approach 2: incubation period split into tertiles		
Incubation period‡		
Less than 1st tertile (shortest)§ (reference group)	1.00	1.00
1st–2nd tertile§	0.55 (0.20–1.48)	0.67 (0.07–3.25)
Greater than 2nd tertile (longest)§	0.26 (0.09–0.91)	0.62 (0.11–3.11)
Age, y	1.12 (1.08–1.16)	1.14 (1.08–1.20)
Sex, M vs. F	2.27 (0.84–7.15)	3.04 (0.91–10.84)

*MERS-CoV, Middle East respiratory syndrome coronavirus; OR, odds ratio.
†Coefficients exp(β) of the logistic regression were estimated by using Markov Chain Monte Carlo methods (10,000 runs) with incubation period as outcome variable and age and sex as predictors. Moreover, 10,000 samples from posterior distributions of incubation periods T for each patient estimated were used in the logistic regression model.
‡10,000 samples of the incubation periods T for each patient were drawn by using Markov Chain Monte Carlo methods.
§Tertiles were 5.1 and 8.0 days for all patients and 5.2 and 8.1 days for patients with exact exposure dates, respectively.

Technical Appendix Table 3. Factors associated with incubation period in fatal and nonfatal cases of infection with MERS-CoV, South Korea*

	Coefficient β (95% CrI)†	
Factor	Fatal cases, n = 36	Nonfatal cases, n = 134
Age	−0.06 (−0.16 to – 0.04)	0.02 (−0.01 to – 0.04)
Sex, M vs. F	−1.02 (−3.38 to – 1.51)	−0.34 (−1.25 to – 0.58)

*MERS-CoV, Middle East respiratory syndrome coronavirus; CrI, credibility interval.
†Coefficients (β) of multiple linear regression were estimated by using Markov Chain Monte Carlo methods (10,000 runs) with incubation period as outcome variable and age and sex as predictors. Moreover, 10,000 samples from posterior distributions of incubation periods T for each patient estimated were used in the multiple regression model.
